# 嵌合抗原受体T细胞序贯异基因造血干细胞移植治疗Ph样急性淋巴细胞白血病21例的疗效及安全性

**DOI:** 10.3760/cma.j.cn121090-20230929-00154

**Published:** 2024-01

**Authors:** 海萍 戴, 宏杰 沈, 正 李, 巍 崔, 庆亚 崔, 梦云 郦, 思帆 陈, 明清 朱, 德沛 吴, 晓文 唐

**Affiliations:** 苏州大学附属第一医院血液内科，国家血液系统疾病临床医学研究中心，江苏省血液研究所，血液学协同创新中心，苏州大学造血干细胞移植研究所，苏州 215006 The First Affiliated Hospital of Soochow University; National Clinical Research Center for Hematologic Diseases; Jiangsu Institute of Hematology; Collaborative Innovation Center of Hematology; Institute of Blood and Marrow Transplantation, Soochow University, Suzhou 215006, China

**Keywords:** 嵌合抗原受体T细胞, 异基因造血干细胞移植, Ph样, 白血病，淋巴细胞，急性, Chimeric antigen T cell, allogenic hematopoietic stem cell transplantation, Ph-like, Acute lymphoblastic leukemia

## Abstract

**目的:**

评估嵌合抗原受体T细胞（CAR-T细胞）序贯异基因造血干细胞移植（allo-HSCT）治疗Ph样急性淋巴细胞白血病（Ph样ALL）患者的疗效及安全性。

**方法:**

纳入2018年3月至2023年 8月在苏州大学附属第一医院接受CAR-T细胞序贯allo-HSCT治疗的21例Ph样ALL患者，对其临床资料进行回顾性分析。

**结果:**

21例患者中，男14例，女7例。接受CAR-T细胞治疗时的中位年龄为22（6～50）岁。7例为ABL1样重排，14例为JAK-STAT重排。接受CAR-T细胞治疗前，12例为血液学未缓解，7例为多参数流式细胞术微小残留病（MFC-MRD）阳性，2例为MFC-MRD阴性。CAR-T细胞均来自患者自体淋巴细胞。9例接受CD19 CAR-T细胞治疗，12例接受CD19/CD22双靶点CAR-T细胞治疗。CAR-T细胞治疗后28 d评估，完全缓解率为95.2％，其中MFC-MRD阴性缓解率75.0％。19例患者出现0～2级细胞因子释放综合征（CRS），2例患者出现3级CRS，经治疗后均恢复。所有患者CAR-T细胞治疗后均接受allo-HSCT。CAR-T细胞治疗后桥接移植的中位时间为63（38～114）d。5例患者CAR-T细胞治疗后出现复发，4例为血液学复发，1例为分子学复发。ABL1组和JAK-STAT组患者的3年总生存率分别为（83.3±15.2）％和（66.6±17.2）％，3年无复发生存率分别为（50.0±20.4）％和（55.6±15.4）％，差异均无统计学意义（*P*值均>0.05）。

**结论:**

CAR-T细胞桥接allo-HSCT，能使大部分Ph样ALL患者迅速达到深度缓解，显著延长患者的无白血病生存。

## Abstract (en)

**Objective:**

To evaluate the efficacy and safety of chimeric antigen receptor T-cell（CAR-T）therapy followed by allogeneic hematopoietic stem cell transplantation（allo-HSCT）in patients with Ph-like acute lymphoblastic leukemia（Ph-ALL）.

**Methods:**

Patients with Ph-ALL who underwent CAR-T therapy followed by allo-HSCT from March 2018 to August 2023 at the First Affiliated Hospital of Soochow University were included, and their clinical data were retrospectively analyzed.

**Results:**

Of the 21 patients, 14 were male and 7 were female. The median age at the time of CAR-T therapy was 22（6–50）years. Seven patients had ABL1-like rearrangements, and 14 had JAK-STAT rearrangements. Prior to CAR-T therapy, 12 patients experienced hematologic relapse; 7 were multiparameter flow cytometry minimal residual disease（MFC-MRD）-positive and 2 were MFC-MRD-negative. CAR-T cells were derived from patients’ autologous lymphocytes. Nine patients were treated with CD19 CAR-T cells, and 12 were treated with CD19/CD22 CAR-T cells. After assessment on day 28 after CAR-T therapy, 95.2% of the patients achieved complete remission, with an MRD-negative remission rate of 75%. Nineteen patients developed grade 0–2 cytokine release syndrome（CRS）and 2 patients suffered grade 3 CRS, all cases of which resolved after treatment. All patients underwent allo-HSCT after CAR-T therapy. The median time from CAR-T therapy to allo-HSCT was 63（38–114）days. Five patients experienced relapse after CAR-T therapy, including four with hematologic relapse and one with molecular relapse. The 3-year overall survival（OS）rates in the ABL1 and JAK-STAT groups were（83.3±15.2）% and（66.6±17.2）%, respectively（*P*＝0.68）. The 3-year relapse-free survival（RFS）rates were（50.0±20.4）% and（55.6±15.4）% in the ABL1 and JAK-STAT groups, respectively. There was no significant difference in 3-year OS or RFS between the two groups.

**Conclusion:**

CAR-T therapy followed by allo-HSCT leads to rapid remission in most patients with Ph-ALL and prolongs leukemia-free survival.

Ph样急性淋巴细胞白血病（ALL）是一类特殊亚型的急性B淋巴细胞白血病（B-ALL），缺乏经典的BCR::ABL1融合基因，但和Ph/BCR::ABL1阳性ALL有高度类似的表达谱特征[1]。Ph样ALL在各年龄段B-ALL中均可见，总体发生率约为20％，但更高发于青少年和年轻成人[2]。Ph样ALL对传统化疗不敏感，采用传统化疗，诱导失败率高，获得缓解的患者多持续微小残留病（MRD）阳性，且易复发，5年总生存（OS）率仅约24％[3]。因此，Ph样ALL在国内外指南中均被归为高危组[4]–[5]。

按照2022年发表的血液肿瘤国际共识分类，Ph样ALL可分为ABL1类重排亚型、JAK-STAT激活亚型和非特指型，前两者最常见[6]。目前已经报道的ABL1 类重排亚型可检测到包括ABL1、ABL2、PDGFRB、CSF1R等基因的重排。国内外多项研究发现，这类患者可能对酪氨酸激酶抑制剂（TKI）敏感[7]。但也有对TKI耐药的病例报道[8]。JAK-STAT激活亚型，包括CRLF2过表达、累及CRLF2的染色体重排、累及JAK2的染色体重排等[6]。JAK抑制剂在伴有JAK-STAT激活的ALL中的安全性和疗效，目前尚无前瞻性临床试验数据披露。但临床前研究结果显示，这类异常可能对JAK抑制剂，如芦可替尼不敏感[9]。因此，这类Ph样ALL 患者缺乏有效的靶向治疗。嵌合抗原受体T细胞（CAR-T细胞）治疗在复发难治B-ALL中可获得80％～90％的缓解率，且研究表明，CAR-T细胞治疗可克服高风险细胞遗传学异常对预后的影响[10]。2017年来，苏州大学附属第一医院陆续采用CAR-T细胞治疗Ph样ALL，现将其中接受过CAR-T细胞并序贯异基因造血干细胞移植（allo-HSCT）治疗的患者资料总结并且报告如下。

## 病例与方法

一、病例

纳入2018年3月至2023年8月在苏州大学附属第一医院接受CAR-T细胞治疗并序贯allo-HSCT的Ph样ALL患者，对其临床资料进行回顾性分析。

二、Ph样ALL的诊断和治疗反应评估

所有患者均进行血常规以及骨髓细胞形态学、免疫表型、染色体核型分析、荧光原位杂交、转录组靶向捕获测序、二代基因测序检查，均符合 Ph 样 ALL 诊断标准[6]。CAR-T细胞回输后第28天评估治疗反应，疗效评估标准及缓解和复发标准参照《中国成人急性淋巴细胞白血病诊断与治疗指南（2021年版）》[5]。采用多参数流式细胞术（MFC）监测MRD，MRD<0.01％定义为MFC-MRD阴性[11]。

三、转录组靶向捕获测序

收集初诊骨髓标本，分离单个核细胞，采用QIAgen公司的RNeasy Mini试剂盒抽提总RNA。取20～100 ng总RNA，选用Illumina公司的TruSeq RNA试剂盒制备RNA 测序文库。选用Illumina NovaSeq平台，对每个样本至少12 G的原始数据进行150 bp读长的成对测序。使用拼接转录物比对软件（STAR, 2.5版）将读数对与人类参考基因组（hg38）进行比对[12]。使用 FusionCatcher 软件查找融合基因。设计针对融合基因的特异性引物，对初诊骨髓标本进行PCR扩增，PCR产物通过一代测序进行验证。

四、CAR-T细胞制备、预处理及回输

采集患者自体外周血淋巴细胞，T细胞功能测试合格后制备CAR-T细胞。CAR-T细胞回输前，采用氟达拉滨、环磷酰胺进行清除淋巴细胞处理。CAR-T细胞按照剂量递增方式回输[13]。细胞因子释放综合征（CRS）分级参照ASTCT标准[14]。

五、allo-HSCT预处理方案和移植物抗宿主病（GVHD）预防

按照苏州大学附属第一医院的常规，选用改良BUCY方案或TBI/CY方案进行移植预处理。单倍型或无关供者移植的GVHD预防均采用抗胸腺细胞球蛋白联合环孢素A、短程甲氨蝶呤以及霉酚酸酯方案。

六、随访

采用查阅门诊/住院电子病例和电话等联系方式进行随访。随访截止时间为2023年9月18日。

七、统计学处理

连续变量的比较采用 Mann-Whitney *U*检验。分类变量的比较采用卡方检验和Fisher精确检验。OS期定义为从CAR-T细胞回输结束到因任何原因死亡或末次随访的时间。无复发生存（RFS）期定义为从CAR-T细胞回输结束到复发或因任何原因死亡的时间。采用Kaplan-Meier法绘制生存曲线，Log-rank检验比较不同组别患者的OS和RFS。*P*<0.05为差异具有统计学意义。采用IBM SPSS 24.0和R 4.0.4软件进行统计分析。

## 结果

一、Ph样ALL的诊断和CAR-T细胞前治疗

入组的21例患者，中位年龄22（6～50）岁。7例患者具有ABL1 class重排，归为ABL1组，其中NUP214::ABL1、ETV6::ABL1、FOXP1::ABL1、NCOR::LYN、EBF1::PDGFRB、TERF2::PDGFRB、KIAA1191::ABL2各1例。13例患者具有JAK-STAT通路激活，归为JAK-STAT组，包括4例P2RY8::CRLF2，5例CRLF2::IgH，4例JAK2重排（STRBP::JAK2 1例，ZBE2::JAK2 1例，RAEBPI::JAK2 1例，PAX5::JAK2 1例）。ABL1组和JAK-STAT组患者初诊时年龄、性别、外周血WBC、骨髓原始细胞比例等方面差异均无统计学意义（表1）。ABL1组5例患者在CAR-T细胞治疗前接受达沙替尼靶向治疗，3例对达沙替尼有治疗反应，2例无治疗反应。JAK-STAT组8例患者在CAR-T细胞治疗前接受芦可替尼靶向治疗，7例对芦可替尼无治疗反应，仅1例对芦可替尼有治疗反应。

**表1:** 21例Ph样急性淋巴细胞白血病（ALL）患者基线特征及CAR-T细胞治疗情况

特征	ABL1组（7例）	JAK-STAT组（14例）	*P*值
年龄［岁，*M*（范围）］	18（6~39）	23（14~50）	0.167
性别［例（%）］			0.354
男	4（57.1）	11（78.6）	
女	3（42.9）	3（21.4）	
初诊WBC［×10^9^/L，*M*（范围）］	47.3（1.8~145.0）	25.0（1.2~217.4）	
CAR-T细胞靶标［例（%）］			1.000
CD19	3（42.9）	6（42.9）	
CD19/CD22	4（57.1）	8（57.1）	
CAR-T细胞治疗前疾病状态［例（%）］			0.089
CR_1_	4（57.1）	4（28.6）	
≥CR_2_	1（14.3）	0（0）	
未缓解	2（28.6）	10（71.4）	
CAR-T细胞治疗后最佳治疗反应［例（%）］			0.635
MRD阴性CR	5（71.4）	10（71.4）	
MRD阳性CR	2（28.6）	3（21.4）	
NR	0（0）	1（7.2）	
CRS分级［例（%）］			0.533
0~2	7（100）	12（85.7）	
3~4	0（0）	2（14.3）	

注 CAR-T细胞：嵌合抗原受体T细胞；MRD：微小残留病；CR_1_：首次完全缓解；≥CR_2_：第2次及以上完全缓解；NR：未缓解；CRS：细胞因子释放综合征

二、CAR-T细胞治疗情况

21例患者中8例为原发难治Ph样ALL，5例为复发Ph样ALL。接受CAR-T细胞治疗前，12例患者处于本病血液学复发状态，9例患者处于血液学缓解状态，其中7例患者MFC-MRD阳性，2例患者MFC-MRD阴性。9例患者接受靶向CD19的CAR-T细胞输注，12例患者接受同时靶向CD19和CD22串联的CAR-T细胞输注。靶向CD19的CAR-T细胞回输量为5×10^6^/kg，同时靶向CD19和CD22串联的CAR-T细胞回输量为（1～2）×10^7^/kg。CAR-T细胞治疗前12例未缓解患者中，经过CAR-T细胞治疗后，11例获血液学缓解，4例MFC-MRD阳性、7例MFC-MRD阴性；CAR-T细胞治疗前9例血液学缓解患者中，经过CAR-T细胞治疗后，1例MFC-MRD阳性，8例MFC-MRD阴性。ABL1组完全缓解（CR）率为100％，MRD阴性CR率71.4％；JAK-STAT组CR率为92.8％，MRD阴性CR率71.4％，两组比较差异无统计学意义（*P*＝0.635）。20例CAR-T细胞治疗后获得治疗反应的患者，中位缓解持续时间为19.3（1.6～62.4）个月。

接受CAR-T细胞治疗后，19例患者出现0～2级CRS，表现为发热、乏力等不适，经对症处理后均缓解。2例患者出现3级CRS，表现为发热、血压下降，依赖小剂量升压药物，经过糖皮质激素、升压等对症处理后均恢复。中位随访29.9（1.6～62.9）个月，ABL1组和JAK-STAT组的3年OS率分别为（83.3±15.2）％和（66.6±17.2）％（*P*＝0.68，图1A），3年RFS率分别为（50.0±20.4）％和（55.6±15.4）％（*P*＝0.69，图1B），差异均无统计学意义。

**图1:**
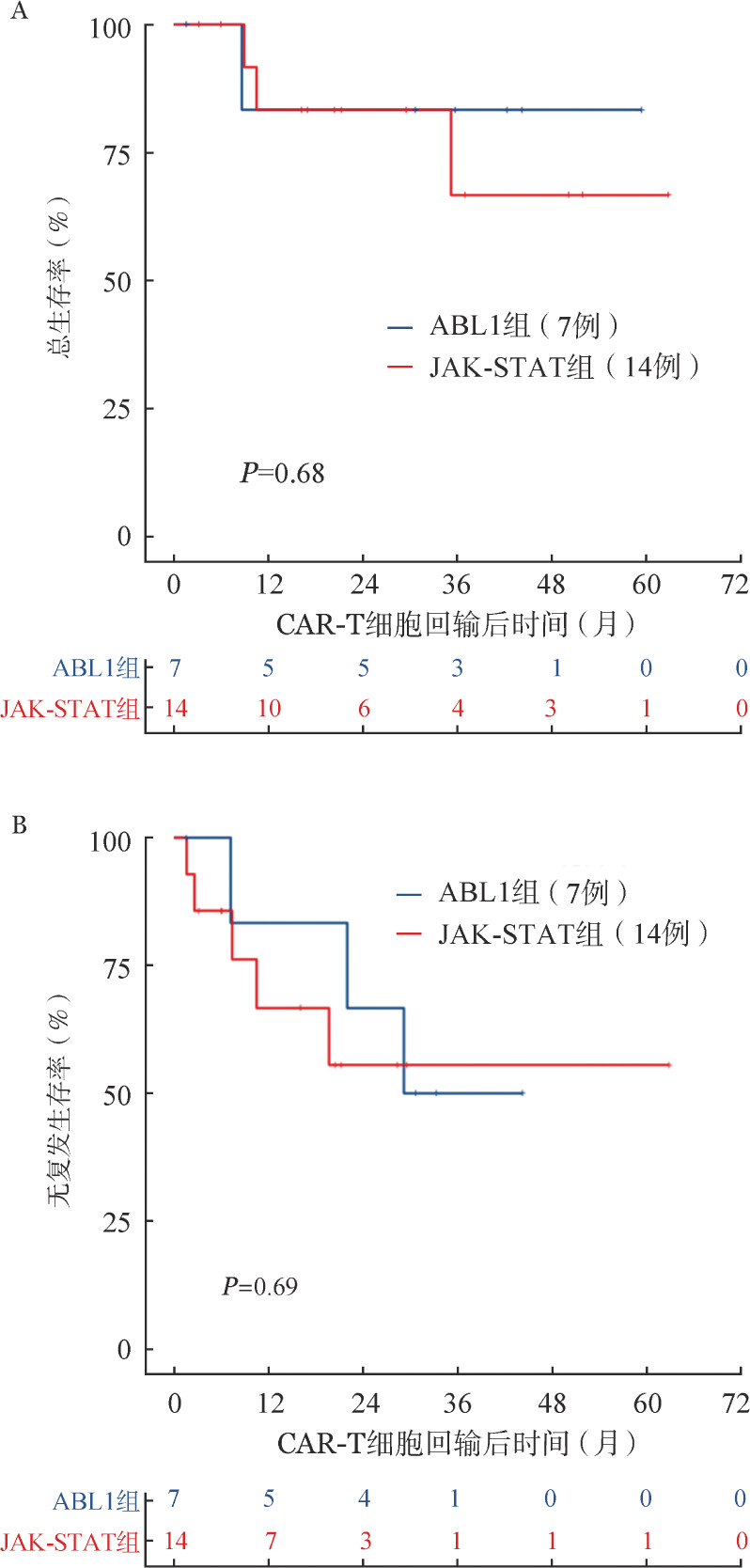
ABL1组和JAK-STAT组Ph样急性淋巴细胞白血病患者总生存曲线（A）和无复发生存曲线（B）

三、allo-HSCT实施情况

21例患者在CAR-T细胞治疗后均接受allo-HSCT，其中5例患者移植前MFC-MRD阳性，1例患者移植前血液学未缓解。CAR-T细胞回输结束至allo-HSCT的中位间隔时间为63（38～114）d。18例患者接受亲缘单倍体移植，3例患者接受无关供者全相合移植。21例患者均接受清髓性预处理，其中18例患者接受改良BUCY方案预处理，2例患者接受TBI/CY方案预处理，1例患者接受白消安、美法仑、克拉屈滨方案预处理。移植后中位粒系造血重建时间为12（10～19）d，中位巨核系造血重建时间为14（10～22）d。移植后30 d复查骨髓，所有患者均获得MFC-MRD阴性缓解。

四、复发和死亡原因

20例对CAR-T细胞有治疗反应的患者，5例（25％）出现复发，复发均发生于移植后，1例为分子学复发，接受贝林妥欧单抗治疗1个周期后再次获得MFC-MRD阴性缓解。4例为血液学复发，其中1例接受供者靶向CD19/CD22串联的CAR-T细胞治疗后获得持续MFC-MRD阴性缓解；1例接受供者靶向CD19的CAR-T细胞治疗后再次获得血液学缓解，但其后迅速出现复发，对贝林妥欧单抗等挽救性治疗无效；1例接受再诱导化疗后获得缓解；1例接受供者淋巴细胞输注后再次缓解，后死于慢性肝脏GVHD。截至末次随访日，4例患者死亡，其中2例死于本病进展，1例死于肝脏GVHD，1例死于CMV肺炎。

## 讨论

据文献报道，TKI等靶向药物单药在复发/难治Ph样ALL治疗中的疗效不佳[1],[15]。本研究中ABL1组5例患者在CAR-T细胞治疗前接受达沙替尼靶向治疗，3例患者接受达沙替尼治疗后获得MRD阳性缓解，其中2例行CAR-T细胞治疗后方获得MRD阴性缓解。JAK-STAT组8例患者在CAR-T细胞治疗前接受芦可替尼靶向治疗，7例对芦可替尼无治疗反应，而这些患者接受CAR-T细胞治疗后，均获得缓解，提示伴有JAK-STAT激活的Ph样ALL，对芦可替尼治疗反应不佳。我们的发现与文献报道一致。上述结果提示，对ABL1重排的Ph样ALL患者，确诊后可尝试TKI治疗，获得血液学缓解后，可以通过细胞免疫治疗清除MRD。对JAK-STAT通路激活的Ph样ALL患者，需尽早规划免疫治疗等非传统治疗方案。

尽管CAR-T细胞治疗的近期有效率高达80％～90％，约50％患者在接受CAR-T细胞治疗后出现复发[16]。另外，非ABL1组的Ph样ALL患者缺乏有效的靶向治疗。据报道，allo-HSCT可减少CAR-T细胞治疗后复发风险[17]。一项回顾性分析中，CAR-T细胞后接受allo-HSCT治疗的复发难治B-ALL患者，4年累积复发率仅11％，4年OS率达70.2％ [18]。一项研究观察了57例Ph样 ALL患者接受allo-HSCT的结果，其中87.7％的患者在allo-HSCT前处于缓解状态，Ph样ALL的无病生存和OS均与标危组相当[19]。上述结果提示，allo-HSCT可以显著改善Ph样ALL患者的生存。

免疫靶向药物，如贝林妥欧单抗、抗CD22的ADC药物奥加伊妥珠单抗，在复发难治B-ALL中取得了令人鼓舞的疗效，为复发难治B-ALL的治疗带来了新选择。由于Ph样ALL发病率低，目前这些药物在Ph样ALL中的疗效和安全性，尚缺乏前瞻性临床研究数据。国内外一系列回顾性研究发现，这些药物在Ph样ALL中也取得了较好的治疗反应。Leahy等[10]的研究中，19例儿童和青少年Ph样ALL接受靶向CD19的CAR-T细胞治疗后，2年OS率达74％。一项来自TOWER研究的事后分析中，15例复发难治Ph样ALL患者，9例接受贝林妥欧单抗再诱导治疗，6例接受传统再诱导化疗。接受贝林妥欧单抗治疗的Ph样ALL患者，总体反应率为46％（CR率36％，CR伴不完全血象恢复率10％），而接受化疗的患者，无一例获得缓解。接受贝林妥欧单抗再诱导治疗的患者，中位OS期为7.9个月，显著长于传统化疗组的4个月[20]。Aldoss等[21]的一项回顾性单中心研究比较了149例至少接受过1个周期新型挽救治疗（包括贝林妥欧单抗、奥加伊妥珠、CD19 CAR-T细胞）的成人复发难治Ph样ALL患者的结局。贝林妥欧单抗、奥加伊妥珠、CD19 CAR-T细胞组的CR/CRi率分别为63％、72％、90％。有治疗反应的患者，接受allo-HSCT巩固治疗，12个月的无事件生存（EFS）率为72％，而在未接受allo-HSCT的患者，12个月的EFS率仅为15％。多因素分析结果也显示，CAR-T细胞治疗后桥接allo-HSCT有助于延长患者生存。

既往指南中，Ph阳性ALL被列为高危组。随着三代TKI以及贝林妥欧单抗等药物在Ph阳性ALL中的应用，该类患者的预后明显改善[22]。目前Ph阳性ALL在部分指南中已经调整为标危组[4]。Ph样ALL在目前发表的国内外指南中仍均被归为高危组。我们的研究中，21例患者接受CAR-T细胞桥接allo-HSCT治疗，3年的OS率和RFS率明显提高，其预后更接近于标危组ALL，与文献报道一致。因此我们推测，随着CAR-T细胞、贝林妥欧单抗、奥加伊妥珠单抗等新型免疫治疗在Ph样ALL中的常规应用，有望改变Ph样ALL的预后分层和治疗结局。

综上所述，CAR-T细胞序贯allo-HSCT治疗Ph样ALL，安全性好，且显著延长患者的RFS期。由于本研究病例数较少，且为回顾性分析，这一策略在Ph样ALL治疗中的长期疗效需要通过开展前瞻性对照临床试验进一步证实。
